# TGF-β1 Exerts Opposing Effects on Grass Carp Leukocytes: Implication in Teleost Immunity, Receptor Signaling and Potential Self-Regulatory Mechanisms

**DOI:** 10.1371/journal.pone.0035011

**Published:** 2012-04-17

**Authors:** Mu Yang, Xinyan Wang, Danyan Chen, Yanan Wang, Anying Zhang, Hong Zhou

**Affiliations:** School of Life Science and Technology, University of Electronic Science and Technology of China, Chengdu, People's Republic of China; National Jewish Health and University of Colorado School of Medicine, United States of America

## Abstract

In fish immunity, the regulatory role of transforming growth factor-β1 (TGF-β1) has not been fully characterized. Here we examined the immunoregulatory effects of TGF-β1 in grass carp peripheral blood leukocytes (PBL) and head kidney leukocytes (HKL). It is interesting that TGF-β1 consistently stimulated the cell viability and the mRNA levels of pro-inflammatory cytokines (*Tnfα* and *Ifnγ*) and T/B cell markers [*Cd4-like (Cd4l)*, *Cd8α*, *Cd8β* and *Igμ*] in PBL, which contrasted with its inhibitory tone in HKL. Further studies showed that grass carp TGF-β1 type I receptor, activin receptor-like kinase 5 (ALK5), was indispensable for the immunoregulatory effects of TGF-β1 in PBL and HKL. Notably, TGF-β1 persistently attenuated ALK5 expression, whereas immunoneutralization of endogenous grass carp TGF-β1 could increase ALK5 mRNA and protein levels. It is consistent with the observation that TGF-β1 decreased the number of ALK5^+^ leukocytes in PBL and HKL, revealing a negative regulation of TGF-β1 signaling at the receptor level. Moreover, transient treatment with TGF-β1 for 24 h was sufficient to induce similar cellular responses compared with the continuous treatment. This indicated a possible mechanism by which TGF-β1 triggered the down-regulation of ALK5 mRNA and protein, leading to the desensitization of grass carp leukocytes toward TGF-β1. Accordingly, our data revealed a dual role of TGF-β1 in teleost immunity in which it can serve as a positive or negative control device and provided additional mechanistic insights as to how TGF-β1 controls its signaling in vertebrate leukocytes.

## Introduction

TGF-β1 is a pleiotropic cytokine that regulates cell development, proliferation, differentiation, migration, and survival in various leukocyte lineages including lymphocytes, dendritic cells, natural killer cells, macrophages and granulocytes [1], [2]. In mammalian immune system, TGF-β1 is a well-known suppressive cytokine and its dominant role is to maintain immune tolerance and suppress autoimmunity [3], [4]. The potent immunosuppressive effects of TGF-β1 are mediated predominantly through its multiple effects on T cells: TGF-β1 suppresses T helper 1 (Th1) and Th2 cell proliferation, while it promotes T regulatory cell generation by inducing Foxp3 expression. On the other hand, TGF-β1 also promotes immune responses by inducing the generation of Th17 cells [1], [5], [6]. Therefore, the regulatory roles of TGF-β1 as a positive or negative control device in immunity are widely acknowledged in mammals [3], [5], [6].

In teleost, despite of lacking extensive investigation on the functional role of TGF-β1, some recent studies have revealed that TGF-β1 also possesses powerful immune depressing actions to the activated leukocytes as that in mammals. For instance, TGF-β1 significantly blocks TNFα-induced activation of macrophage in goldfish and common carp, but it induces the proliferation of goldfish fibroblast cell line CCL71 [7], [8]. In grass carp, TGF-β1 down-regulates LPS/PHA-stimulated the proliferation of peripheral blood lymphocyte by contrast with the stimulatory effect of TGF-β1 alone in the same cells [9]. In red sea bream, similar phenomenon was observed during leukocyte migration under TGF-β1 treatment with or without LPS challenge [10]. These findings not only define the TGF-β1 as an immune regulator in teleost, but also indicate that TGF-β1 may retain similar functions in immunity during the evolution of vertebrates.

Activated mammalian TGF-β1 initiates its downstream signaling events by activating two types of transmembrane serine/threonine kinase receptors classified as type II (TGF-β1 type II receptor, TβRII) and type I (TβRI, also termed as ALK5). In mammals, ALK5 and TβRII are specific for TGF-β1 and mediate its most cellular responses [11]. Considering the immunoregulatory role of TGF-β1, the involvement of ALK5 in immune regulation has been preliminarily defined. A recent study reported that ALK5 was critical to the thymic development of natural CD4^+^CD25^+^Foxp3^+^ T cells [12]. However, it is unclear whether ALK5 expression level is associated with TGF-β1 regulation of immune responses. In teleost, only zebrafish *Alk5* cDNA is available in GenBank, and the feature and expression of ALK5 in teleost still remain unknown. In this study, we isolated and identified grass carp *Alk5* cDNA from head kidney. Tissue distribution assay showed that *Alk5* mRNA was highly expressed in grass carp peripheral blood leukocytes (PBL) and head kidney leukocytes (HKL), indicating that TGF-β1 may play a role in these cell models. Interestingly, our results demonstrated that TGF-β1 exerted opposing immunomodulatory effects on grass carp PBL and HKL. Furthermore, we found that similar to the mammalian system, ALK5 was required for the immunoregulatory effects of TGF-β1 in grass carp leukocytes. Notably, TGF-β1 persistently down-regulated ALK5 expression at both mRNA and protein levels in these two cell groups. These studies provide new insights into the regulatory role of TGF-β1 in fish immune system and more understanding of TGF-β1 signaling control in teleost.

## Results

### TGF-β1 exhibits opposite regulatory effects on PBL and HKL

**Figure 1:**
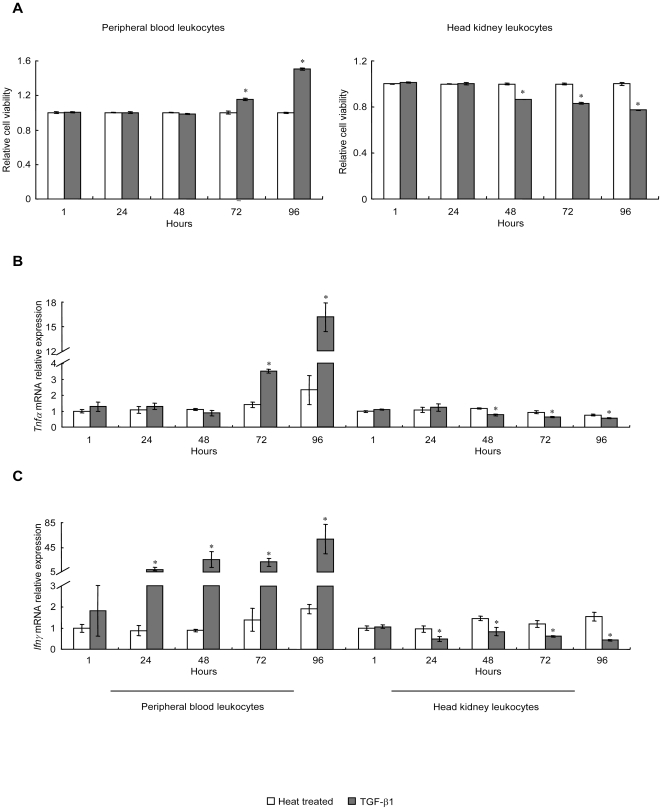
Immunoregulatory effects of TGF-β1 on the cell viability and immune-related gene mRNA expression in grass carp PBL and HKL. A, Leukocytes were incubated with 100 ng/ml of native or heat treated rgcTGF-β1 for 1, 24, 48, 72 and 96 h. The viability of PBL (A, left panel) and HKL (A, right panel) was detected by CCK-8 assay. Relative cell viability was expressed as the fold changes of the respective heat treated group at each time point. B–G, Effects of rgcTGF-β1 on *Tnfα* (B), *Ifnγ* (C), *Cd4l* (D), *Cd8α* (E), *Cd8β* (F) and *Igμ* (G) mRNA expression. Grass carp PBL or HKL were incubated with 100 ng/ml of native or heat treated rgcTGF-β1 for the duration as indicated. Results from PBL are presented in the left panels and results from HKL are presented in the right panels. qPCR was carried out to determine the mRNA expression profiles of these genes as described in Materials and methods. Relative mRNA levels were analyzed by using *β-actin* as an internal reference and expressed as the fold changes of the heat treated group at 1 h. Data presented (mean±SEM, *N* = 4) are representative results of three individual experiments. The asterisk (*) denotes a significant difference at P<0.05.

Recombinant grass carp TGF-β1 (rgcTGF-β1) was prepared by using the pET 30a(+) prokaryotic expression system. The endotoxin level in the purified rgcTGF-β1 was determined, showing that the LPS content in the rgcTGF-β1 was very low, typically below 0.7 EU in the 100 ng of rgcTGF-β1. After that, a dose-dependent experiment was performed, showing that treatment of 25–400 ng/ml of rgcTGF-β1 stimulated or inhibited the cell viability of PBL and HKL, respectively. Notably, both stimulation and inhibition of rgcTGF-β1 reached a stable phase at the doses higher than 100 ng/ml (**Fig. S1A**). Meanwhile, grass carp PBL and HKL were treated with 100 ng/ml of rgcTGF-β1 for 1, 24, 48, 72 and 96 h. The CCK-8 assay showed that TGF-β1 enhanced the cell viability of PBL from 72 to 96 h when compared with the time-matched controls (heat treated rgcTGF-β1 group) (**Fig. 1A** **, left panel**). In contrast, rgcTGF-β1 reduced HKL viability from 48 to 96 h (**Fig. 1A** **, right panel**). As a control, recombinant grass carp squint (rgcSQT), another member of TGF-β superfamily, was prepared by the same method for rgcTGF-β1 preparation following our previous study [13]. Results showed that treatment with 100 ng/ml of rgcSQT for 72 h was no effect on both PBL and HKL viability (Fig. 1A, *small inset*), suggesting the specificity of rgcTGF-β1 on PBL and HKL. In parallel experiments, trunk kidney leukocytes (TKL) were incubated with increasing doses (25–400 ng/ml) of rgcTGF-β1 for 72 h, showing that rgcTGF-β1 at the doses higher than 100 ng/ml was effective but minor in elevating the cell viability of TKL (**Fig. S1B**). To further elucidate the immunoregulatory effects of TGF-β1 on these two cell groups, the mRNA levels of inflammatory cytokines (*Tnfα* and *Ifnγ*) (**Fig. 1B, C**) and T/B lymphocyte markers (*Cd4l*, *Cd8α*, *Cd8β* and *Igμ*) (**Fig. 1D, E, F, G**) were determined by qPCR. Interestingly, rgcTGF-β1 had stimulatory or inhibitory effects on *Tnfα*, *Ifnγ Cd4l*, *Cd8α* and *Igμ*mRNA expression in PBL (**Fig. 1B, C, D, E, G** **, left panels**) and HKL (**Fig. 1B, C, D, E, G** **, right panels**), respectively. However, rgcTGF-β1 significantly stimulated *Cd8β* expression in PBL (**Fig. 1F** **, left panel**) but had no effect on this gene in HKL (**Fig. 1F** **, right panel**). Obviously, rgcTGF-β1 showed consistent stimulatory effects in PBL in contrast to its inhibitory tones in HKL at both cell viability and immune relevant gene expression.

### ALK5 was required for the immunoregulatory effects of TGF-β1 in PBL and HKL

**Figure 2:**
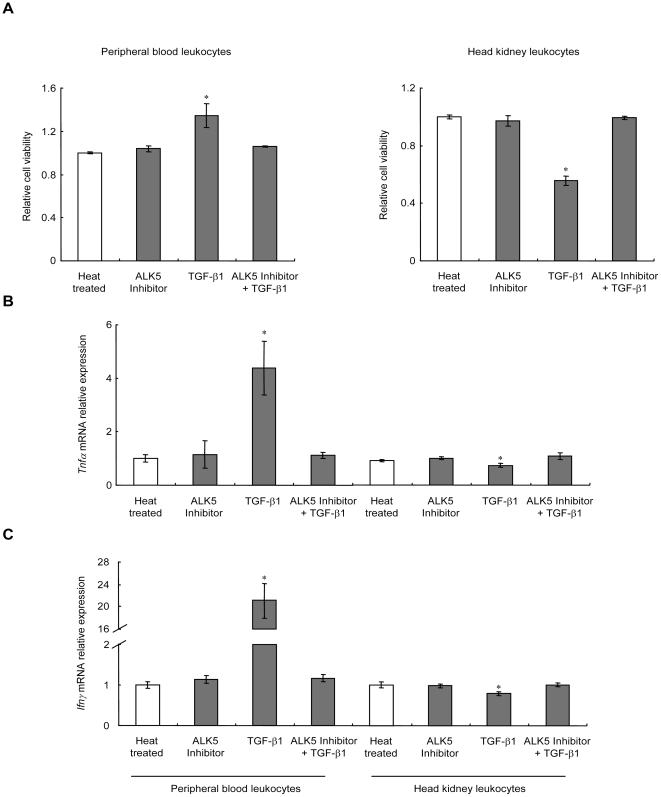
Effect of ALK5 inhibitor on TGF-β1-induced immune responses. Grass carp PBL and HKL were exposed to 100 ng/ml of native or heat treated rgcTGF-β1 in the presence or absence of 2 µM of ALK5 inhibitor for 72 h under static incubation. After that, the viabilities of PBL or HKL were measured by CCK-8 assay (A). The relative cell viability was expressed as the fold changes of the heat treated group. qPCR was performed to detect mRNA levels of *Tnfα* (B), *Ifnγ* (C), *Cd4l* (D), *Cd8α* (E), *Cd8β* (F) and *Igμ* (G) as described in Materials and methods. Results from PBL are presented in the left panels and results from HKL are presented in the right panels. Data presented (mean±SEM, *N* = 4) are representative results of three individual experiments. The asterisk (*) denotes a significant difference at P<0.05.

To examine the role of ALK5 in TGF-β1-induced cell viability and lymphocyte marker gene expression, grass carp PBL and HKL were exposed to rgcTGF-β1 (100 ng/ml) for 72 h in the presence or absence of ALK5 inhibitor (2 µM). Compared with control group (heat treated rgcTGF-β1), treatment with rgcTGF-β1 alone consistently affected the cell viability and mRNA expression of the genes (*Tnfα*, *Ifnγ*, *Cd4l*, *Cd8α*, *Cd8β* and *Igμ*) as described above (**Fig. 2 A, B, C, D, E, F, G**). These stimulatory action in PBL (**Fig. 2 A–G** **, left panels**) and inhibitory action in HKL (**Fig. 2 A, B, C, D, E, F, G** **, right panels**), however, were blocked by ALK5 inhibitor. In these experiments, parallel treatment with ALK5 inhibitor (2 µM) alone had no effect on the cell viability or mRNA expression of the genes. In addition, the minimal dose of the ALK5 inhibitor tested to induce maximal inhibition to TGF-β1 treatment was noted at 2 µM in both PBL and HKL (**Fig. S1C**).

### Molecular cloning and sequence analysis of grass carp *Alk5*

We obtained the full-length cDNA sequence of grass carp *Alk5* (Accession no: HM356028), which consisted a 46 bp 5′ UTR, a 67 bp 3′ UTR, and a 1503 bp ORF encoded a 500-aa polypeptide (**Fig. S2A**). Meanwhile, the 56 kDa putative grass carp ALK5 protein was analyzed to identify the characteristic domains, including a glycine/serine-rich motif (GS motif) and a serine/threonine protein kinase domain. Moreover, two highly conserved regions, TSGSGSG and HRDLKSKN, were revealed in the GS motif and serine/threonine kinase domain, respectively (**Fig. S2B**). Based on the Neighbor-Joining method, a phylogenetic tree was constructed to reveal the evolutionary relationship of grass carp ALK5 with their counterparts in other vertebrates (**Fig. S2C**). The deduced amino acid sequence exhibited 91.6% of identity to zebrafish ALK5 and more than 75% identity to other vertebrate counterparts, indicating that this receptor is highly conserved during evolution.

### Tissue distribution of grass carp *Alk5*

**Figure 3:**
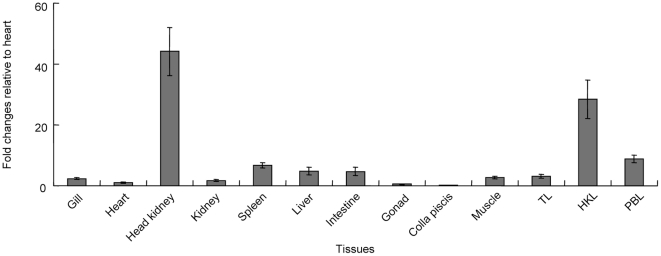
Expression pattern of grass carp *Alk5* mRNA in various tissues and leukocytes. Total RNA were extracted from the selected tissues or leukocytes, and the mRNA levels of *Alk5* and *β-actin* were assessed by qPCR as described in Materials and methods. The *alk5* mRNA levels were calibrated by *β-actin* in the same samples and expressed as the fold changes of the level in heart. Data presented (mean±SEM, *N* = 4) are pooled results from four independent fishes. TL, thymus leukocytes; HKL, head kidney leukocytes; PBL, peripheral blood leukocytes.

Grass carp *Alk5* mRNA was expressed in all selected tissues, except heart, gonad and colla piscis. The highest mRNA levels were detected in head kidney, to lesser extent in spleen, liver and intestine, to low levels in gill, kidney, muscle and thymus leukocytes (TL). Notably, relatively high abundance of *Alk5* transcript was detected in HKL and PBL (**Fig. 3**).

### Validation of the specificity of antibodies for grass carp TGF-β1 and ALK5

Using the total proteins from grass carp PBL and HKL, mouse monoclonal Ab for grass carp TGF-β1 (gcTGF-β1 mAb) could specifically recognized a protein with 13 kDa (**Fig. S3, left panel**), which is corresponding to the predicated molecular weight of grass carp TGF-β1. In addition, the specificity was verified by antibody preabsorption with 100 µg of rgcTGF-β1 (**Fig. S3, right panel**). For grass carp ALK5, alignment of the deduced amino acid sequences revealed that the structural characteristics, in particular those in GS motif and kinase domain, were highly conserved in ALK5 from fish to mammal (**Fig. S2B**). This supports the notion that the commercial antibody (rabbit polyclonal Ab to human ALK5, ALK5 pAb, Abcam) which is widely used in detection of mammalian, amphibious and chicken ALK5 (referred to the product manual) may specifically interact with grass carp ALK5. In fact, WB in this study showed a 56 kDa single band of ALK5 immunoreactivity in both grass carp PBL and HKL (**Fig. S4A**). Moreover, the specificity of this antibody was confirmed by ICC assay (**Fig. S4B**), showing that ALK5^+^ cells were present in both PBL and HKL, whereas no positive signal was detected in the isotype groups.

### Effect of TGF-β1 on ALK5 mRNA and protein levels in PBL and HKL

**Figure 4:**
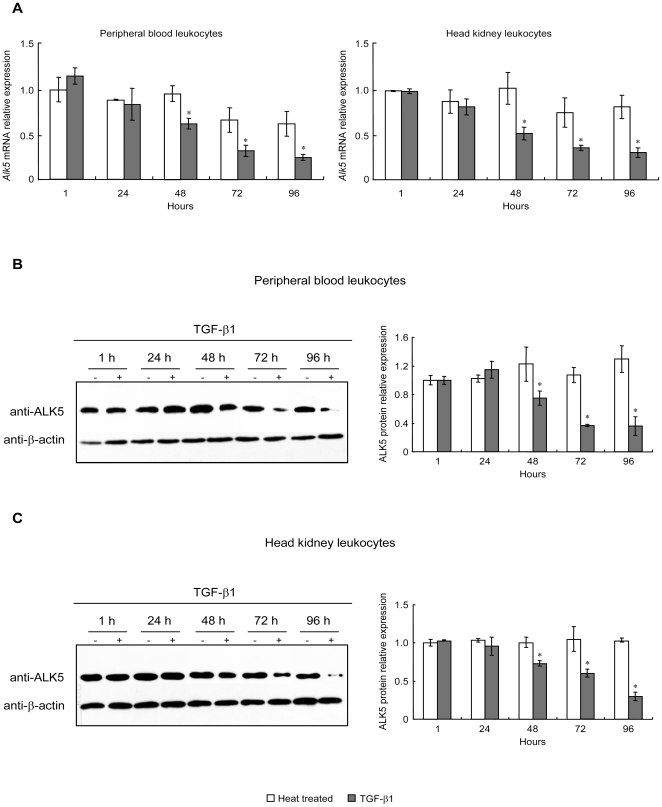
Effects of TGF-β1 on ALK5 mRNA and protein levels in PBL and HKL. After treatment with native or heat treated rgcTGF-β1 (100 ng/ml) for 1–96 h, ALK5 mRNA (A) and protein (B–C) levels in PBL or HKL were analyzed by qPCR and WB, respectively. In parallel experiments, leukocytes were exposed to the decreasing dilution of gcTGF-β1 mAb (1∶30000-1∶300) for 72 h under static incubation. Subsequently, qPCR and WB were performed for detection of ALK5 mRNA expression (D) and protein levels (E) in PBL (left panels) or HKL (right panels). Relative mRNA expression levels of *Alk5* were analyzed using *β-actin* as an internal reference and expressed as the fold changes of the heat treated group at 1 h. Data presented (mean±SEM, *N* = 4) are representative results from three individual experiments. In WB, the representative results were showed here and β-actin levels were used as an internal control and isotype control was mouse IgG (30 µg/ml). Meanwhile, the densitometric analysis of ALK5 protein levels was performed (mean±SEM, *N* = 4) and the relative protein levels were expressed as the fold changes of the heat treated group at 1 h. The asterisk (*) denotes a significant difference at P<0.05.

To investigate whether TGF-β1 affect its type I receptor mRNA and protein levels, PBL and HKL were treated with 100 ng/ml of rgcTGF-β1 from 1 to 96 h. After that, qPCR and WB were performed to detect mRNA and protein levels of ALK5, respectively. Interestingly, TGF-β1 consistently down-regulated both mRNA (**Fig. 4A**) and protein (**Fig. 4B, C**) levels of ALK5 in PBL and HKL after 48 h treatment. However, a 72 h-incubation of TKL with 100 ng/ml of rgcTGF-β1 did not affect ALK5 mRNA and protein expression (**Fig. S5A, B**). To further test whether endogenous TGF-β1 released from PBL and HKL could modify the expression of ALK5, TGF-β1 immunoneutralization by using gcTGF-β1 mAb was carried out to neutralize the secreted TGF-β1 in cell culture. Results showed that 72 h-incubation of PBL or HKL with decreasing dilution of TGF-β1 mAb (1∶10000-1∶300) was effective in elevating ALK5 mRNA (**Fig. 4D**) and protein levels (**Fig. 4E**). In these experiments, treatment with mouse IgG, the isotype control for TGF-β1 immunoneutralization, was not effective in altering ALK5 mRNA and protein levels in both PBL and HKL (**Fig. 4D, E**).

### TGF-β1 reduced the proportion of ALK5^+^ leukocytes in PBL and HKL

**Figure 5:**
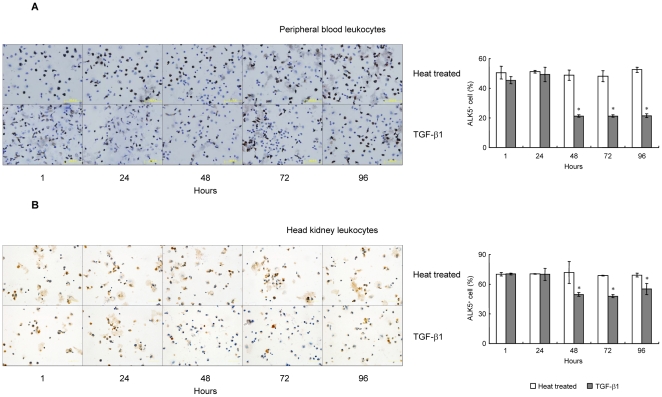
ICC staining and statistical analysis of ALK5^+^ cells in grass carp PBL and HKL. Grass carp PBL (A) and HKL (B) were treated with 100 ng/ml of rgcTGF-β1 for 1, 24, 48, 72 and 96 h, and subsequently fixed on coverslips. After that, ICC staining was preformed by using the ALK5 pAb and ALK5^+^ cells were observed by using phase contrast microscopy (×400). Representative ICC staining of PBL or HKL is presented in the left panels and the statistical analysis of the numbers of ALK5^+^ cells in PBL or HKL is shown in the right panels. The number of ALK5^+^ cells was shown as the percentage of the amount of cells and presented as mean±SEM (*N* = 4). The asterisk (*) denotes a significant difference at P<0.05.

To further clarify the details of TGF-β1 inhibition on ALK5 expression in grass carp PBL and HKL, effect of TGF-β1 on the proportion of ALK5^+^ leukocytes was assessed by ICC. In this case, a time-course study was conducted by static incubation of PBL or HKL with 100 ng/ml of rgcTGF-β1 for 1, 24, 48, 72 and 96 h (**Fig. 5**). Within 24 h, TGF-β1 did not affect the proportion of ALK5^+^ leukocytes in both PBL (**Fig. 5A**) and HKL (**Fig. 5B**) when compared with the time-matched controls. However, a significantly decline of the number of ALK5^+^ leukocytes was consistently observed from 48 to 96 h in both cell groups (**Fig. 5**). In parallel experiments, however, 100 ng/ml of rgcTGF-β1 was not effective in altering the proportion of ALK5^+^ in TKL (**Fig. S5C**).

### Triggered TGF-β1 signaling is sufficient to maintain cellular responses in PBL and HKL

**Figure 6:**
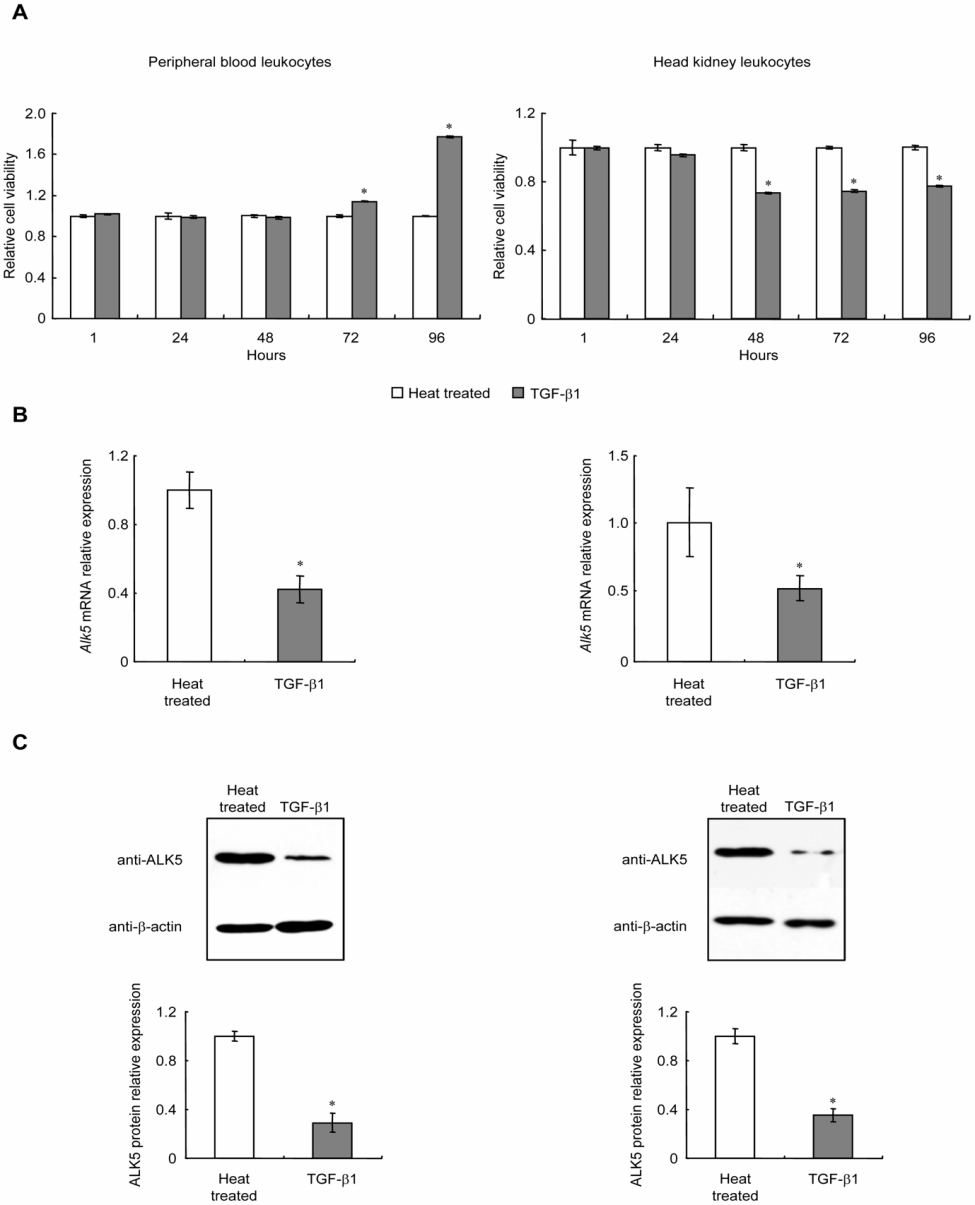
Effects of transient treatment with rgcTGF-β1 for 24 h on cell viability and ALK5 expression in grass carp PBL and HKL. In this study, treatment with 100 ng/ml of native or heat treated rgcTGF-β1 was carried out for 24 h and subsequently terminated by substitution of fresh medium without drug. After that, the cells were incubated for additional 24, 48 and 72 h. Time course effects of transient treatment with TGF-β1 for 24 h on cell viability of PBL and HKL were shown (A). Relative cell viability was expressed as the fold changes of the respective heat treated group at each time point. Data presented (mean±SEM, *N* = 4) are representative results of three individual experiments. In addition, the ALK5 mRNA (B) and protein (C–D) levels in PBL (left panels) or HKL (right panels) with additional 48 h incubation after TGF-β1 transient treatment were determined. Relative mRNA expression and protein levels of ALK5 were analyzed by using *β-actin* as an internal reference and expressed as the fold changes of the heat treated group. Data presented (mean±SEM, *N* = 4) are representative results from three individual experiments. Control, medium was replaced with fresh one after 24 h incubation; TGF-β1, TGF-β1 treatment for 24 h was terminated by fresh medium. The asterisk (*) denotes a significant difference at P<0.05.

To assess whether TGF-β1 could trigger these cellular responses, PBL and HKL were transiently treated with rgcTGF-β1 (100 ng/ml) for 24 h. Cell viabilities were determined at 24, 48 and 72 h after removal of TGF-β1. As shown in **Fig. 6A**, similar to the results with the continuous treatment for 96 h in PBL and HKL (**Fig. 1A**), the changes of PBL (left panel) and HKL (right panel) viabilities induced by TGF-β1 were still observed at the same initial time point (72 h or 48 h) in transient treatment experiments. In parallel experiments, transient treatment with TGF-β1 for 24 h in grass carp PBL and HKL significantly down-regulated ALK5 mRNA expression (**Fig. 6B**) and protein levels (**Fig. 6C, D**) after the cells were incubated for additional 48 h, which was in agreement with the results of continuous treatment with TGF-β1 for 72 h in **Fig. 4A, B, C**. Thus, transient treatment with TGF-β1 for 24 h was sufficient to induce similar cellular responses during the prolonged incubation period compared with continuous treatment.

## Discussion

In this study, we investigated the effects of TGF-β1 on grass carp leukocytes. Our results showed that administration of TGF-β1 increased PBL viability, but inhibited HKL viability. Consistent with this observation, TGF-β1 also exhibited its opposing effects in *Tnf*α and *Ifnγ* expression in PBL and HKL, suggesting that TGF-β1 mediated immune responses in fish leukocytes at least partly via modulating the expression of pro-inflammatory cytokines. As in mammals, TNFα is defined to be a central effector cytokine in inflammatory and anti-microbial responses in teleost [14], [15], [16], [17], while IFNγ is another classical pro-inflammatory cytokine and its function has been recently identified in some fishes [18], [19], [20], [21], in which IFNγ promotes the phagocytic and antiviral activities as its mammalian counterparts. In this view, the importance of TNFα and IFNγ in fish immunity has been established, which supported the role of TGF-β1 as a regulator for controlling the production of pro-inflammatory cytokines in grass carp immune system. Another notable finding is that TGF-β1 showed stimulatory or inhibitory effects on the mRNA expression of T/B cell markers (*Cd4l*, *Cd8α*, *Cd8β* and *Igγ*) in different grass carp leukocyte groups. This observation seems to support the hypothesis that TGF-β1 may regulate the status of grass carp T/B cell. In support of this notion, it has previously been reported that CD8α/CD8β expression corresponds to cytotoxic lymphocyte activity in carp [22], [23], allogeneic stimulation in rainbow trout [24], as well as virus infection in salmon [22], [24], [25]. Additionally, the expression patterns of *Cd4* and *Cd8α* have been applied to serve as the markers for T lymphocyte differentiation in sea bass [26]. Meanwhile, it has been demonstrated that *Igμ* expression is associated with the activation of B lymphocytes in many fish species [9], [27], [28], [29]. Taken together, our results indicated that TGF-β1 may affect T/B lymphocyte activation and functionality in grass carp PBL and HKL.

As noted above, TGF-β1 displayed its dualistic nature at both cellular and molecular levels in grass carp PBL and HKL. Since the precise action of TGF-β1 is determined by cell type, cell state and local cellular environment in mammal [2], we hypothesized that the bidirectional function of TGF-β1 in teleost may be subject, at least in part, to the different type and/or status of those subsets in grass carp PBL and HKL. In fact, staining assay by using hematoxylin-eosin and Sudan black B showed that lymphocytes were enriched in grass carp PBL (≥94%) and HKL (≥85%) (Data not shown). Therefore, PBL contained more lymphocytes than that of HKL, indicating the difference of subpopulation composition in these two cell groups. This difference was also reported in sea bream [30] and salmon [31] because the fraction of subpopulations recognized by mAb/pAb was obviously different between PBL and HKL. Consistently, the proportion of B cells in PBL was significantly different from that in HKL in rainbow trout [32], common carp [33] and Atlantic cod [34]. In addition, we also found that rgcTGF-β1 was effective but minor in elevating the cell viability of grass carp TKL (**Fig. S1B**), indicating that the activity of TGF-β1 may be subject to cell populations. In support of this notion, 100 ng/ml of rgcTGF-β1 did not affect ALK5 expression and ALK5^+^ cell proportion in TKL (**Fig. S5**), which were different from the findings in PBL and HKL (**Fig. 4** **and** **Fig. 5**). Besides the difference of subsets, grass carp PBL and HKL also displayed different immunological status in view of the facts that TGF-β1 induced opposite effects on mRNA expression of grass carp T/B cell markers in these two cell groups. Moreover, the expression patterns of these marker genes in grass carp leukocytes correlated well with the effects of TGF-β1 on cell viabilities, implying the function polarity of TGF-β1 in distinct leukocytes (**Fig. 1**). These findings prompted us to speculate that PBL may have immunological characteristics opposite to that of HKL. Evidence in support of this hypothesis is the existence of different development or maturation status of subpopulation from fish PBL and HKL. Upon LPS induction, the immune cells from trout kidney have great capacity for the generation of plasmablasts and plasma cells, but those from blood which mainly houses resting B cells can not differentiate into plasma cells, suggesting there were more highly proliferative cells in kidney than in blood [32]. In common carp, PBL contained most probably monocytes, whereas the cells from HKL are most likely to be enriched in adult macrophages [33], [35], indicating the different maturation status of macrophages in carp PBL and HKL. Interestingly, a recent review summarized that both CD4 and CD8 showed higher expression levels in head kidney than peripheral blood in several fish species, implying the different activation status of T cells in fish PBL and HKL [27]. Taken together, it seems that the bidirectional function of TGF-β1 in grass carp may correlate with the opposite status of the subsets in PBL and HKL.

To our knowledge, the functional role of ALK5 in fish still remains unknown, and even no information about ALK5 expression and regulation is available in fish. In this study, we found that grass carp *Alk5* was predominantly distributed in head kidney and HKL, and in PBL with relatively high abundance. This finding prompted us to investigate the role of ALK5 in the TGF-β1-induced immune responses. Our data showed that grass carp ALK5 is indispensable for TGF-β1 actions in both HKL and PBL, which was consistent with the functional role of ALK5 in mammals. More interestingly, TGF-β1 induced a persistently reduction of mRNA and protein levels of ALK5 in grass carp PBL and HKL (**Fig. 4A, B, C**). The response was confirmed by the immunoneutralization experiments in which neutralization of endogenously secreted TGF-β1 significantly enhanced ALK5 mRNA and protein levels in both leukocyte groups (**Fig. 4D, E**). Furthermore, ICC assay showed TGF-β1 also decreased the proportions of ALK5^+^ leukocytes in both PBL and HKL (**Fig. 5**), implying that ALK5 degradation/endocytosis was induced by TGF-β1. This is the first time to present the evidence that TGF-β1 has a potent self-regulatory characteristic on its receptor expression in fish, supporting the concept that ALK5 may serve as a local regulatory point involving in the immune response of grass carp.

In agreement with our findings, it has become evident that mammalian TGF-β1 can cause its receptor ubiquitylation and subsequent degradation/endocytosis in various cells [36], [37], [38]. In human, repression of TGF-β receptor expression is considered as a common mechanism that enables tumor cells to develop a resistance to growth inhibition induced by TGF-β1 [39]. With respect to immunity, an early study reported that down-regulation of ALK5 was shown to correlate to a diminished ability of human monocytes to the proinflammatory effects of TGF-β [40]. Collectively, it is therefore conceivable that there may be a negative regulation mechanism at the receptor level to limit the magnitude of TGF-β1 signaling in grass carp PBL and HKL, further to prevent excessive responses in immune cells. Notably, we found that these cellular responses described above did not require persistent TGF-β1 stimulation (**Fig. 6**), indicating that one possible mechanism by which TGF-β1 triggered down-regulation of ALK5 protein and mRNA expression, leads to the desensitization of the leukocytes toward TGF-β1 stimulation.

Overall, our data revealed a dual role of TGF-β1 in teleost immunity in which it can serve as a positive or negative control device and provided additional mechanistic insights to how TGF-β1 controls its signaling in vertebrate leukocytes.

## Materials and Methods

### Animals

One-year-old Chinese grass carp (*Ctenopharyngodon idellus*), weighing from 1 to 1.5 kg, were obtained from Chengdu Tongwei Aquatic Science and Technology Company (Chengdu, CH). The fish was maintained in laboratory with natural temperature and photoperiod for 2 weeks prior to experimental processing. All experiments complied with the Regulation of Animal Experimentation of Sichuan province, China.

### Preparation of recombinant grass carp TGF-β1

The cDNA sequence encoding the mature grass carp TGF-β1 (amino acid residues 266–377) was amplified using the primers shown in Table S1 (tF and tR). The sequence was cloned to pET30a (Novagen, EMD Chemicals, Madison, USA) vector to obtain the construct of pET30-TGF-β1. Subsequently, the plasmid was transformed into BL21 (DE3) (Invitrogen, Carlsbad, USA) competent cells. The cells were cultured in 200 ml of LB medium containing 100 µg/ml of ampicillin at 25°C with shaking at 180 rpm and 1 mM isopropyl-1-thio-β-D-galactopyranoside (IPTG) (Merck, Gmbh, DE) was added to the culture for additional 4 h during exponential growth. The cells were harvested by centrifugation at 3,000 *g* at 4°C for 10 min and resuspended in binding buffer (20 mM imidazole, 500 mM NaCl, and 20 mM PB, pH 8.0) supplemented with the EDTA-free Protease Inhibitor Cocktail Tablets (Roche, Indianapolis, USA). The suspension was sonicated and the cell lysate was clarified by centrifugation at 10,000 *g* for 20 min at 4°C. Then supernatant was loaded in a HisTrap column (GE Healthcare, Piscataway, USA). The bound protein was eluted by elution buffer (500 mM imidazole, 500 mM NaCl, 20 mM PB, pH8.0) and desalted with a HiTrap Desalting Column (GE Healthcare) in 10 mM citric acid buffer (pH 3.0) to obtain the His-tagged TGF-β1. All the affinity chromatography and desalting proceedings were run on ÄKTAexplorer 100 (GE Healthcare). The Bradford protein assay (Bio-Rad) was carried out to determine the protein concentrations. The endotoxin levels in the purified protein were determined by using an endotoxin assay kit (Chinese Horseshoe Crab Reagent Manufactory, Xiamen, CH).

### Preparation of grass carp TL, HKL, TKL and PBL

Grass carp TL, HKL, TKL and PBL were prepared by density gradient centrifugation referring to the method of grass carp HKL isolation with some modifications [41]. Briefly, head kidney, trunk kidney or thymus was removed from grass carp and washed twice by D'HANK's balanced salt solution (HBSS, Sigma-Aldrich, Egham, UK). The cell suspensions were obtained by macerating the head kidney, trunk kidney or thymus on stainless steel mesh with the help of pestle in HBSS. Peripheral blood was obtained from cardiac atrium of grass carp using a heparinized syringe. The cell suspensions from each organ were layered over a discontinuous gradient of fish leukocytes isolation medium (WBC1080F, TBD, Tianjin, CH) with equal volume. After centrifugation (30 min at 1300 g) at room temperature, the cells at the interface were collected and washed twice (15 min at 900 *g*) at 4°C by RPMI-1640 medium (GIBCO, Grand Island, USA). The cells were resuspended in RPMI-1640 medium supplemented with 10% FBS (PAA, Gmbh, DE) for further use. Freshly prepared PBL, HKL or TKL were cultured in 24-well plate (Becton Dikinson, Franklin Lakes, USA) at a seeding density of approximately 6×10^5^ cells/ml/well and incubated overnight (>15 h) at 27°C under 5% CO_2_ and saturated humidity. On the following day, drug treatment with native or heat treated rgcTGF-β1, gcTGF-β1 mAb, which was prepared via custom antibody services (Abmart, Shanghai, CH), rgcSQT or TGF-β RI Kinase inhibitor VIII (ALK5 inhibitor, Calbiochem, EMD Chemicals, San Diego, USA) was initiated for the duration as indicated in individual experiments.

### Cloning and sequence analysis of grass carp *Alk5*

Five µg of total RNA was extracted from grass carp HKL using TRIzol Reagent (Invitrogen) and reverse-transcribed into cDNA using Superscript III reverse transcriptase (Invitrogen). The DNA fragment for *Alk5* was obtained by PCR amplification with the degenerated primers (alkF and alkR, Table S1) and subcloned into pGM-T vector (TaKaRa, Dalian, CH) for sequencing. Based on the partial sequences, gene-specific primers (alk5p1, alk5p2, alk3p1 and alk3p2) were designed (Table S1), and 3′/5′-RACE were performed using the GeneRacer Kit (Invitrogen). The PCR products were gel-purified and subcloned into pGM-T vector for DNA sequencing. Both the open reading frame and the deduced protein sequence of *Alk5* were analyzed by ORF Finder (http://www.ncbi.nlm.nih.gov/projects/gorf/). Following the similarity analysis by using nucleotide and protein BLAST programs from NCBI, multiple alignments were carried out by using DNAMAN software (Lynnon Biosoft, Quebec, CA). Characteristic motifs were predicted by ScanProsite (http://www.expasy.ch/tools/scanprosite/). Phylogenetic trees were constructed using the Neighbor-Joining method by Molecular Evolutionary Genetics Analysis (MEGA) 3.1 with bootstrap value of 1,000.

### Specificity analysis of the antibodies for grass carp TGF-β1 and ALK5

The TGF-β1 mAb was prepared via custom antibody services (Abmart), and its specificity was determined by antibody preabsorption using WB analysis. In this case, The PVDF membrane with PBL and HKL total protein was incubated with gcTGF-β1 mAb in the presence of excessive rgcTGF-β1. Similarly, the specificity of commercial rabbit pAb to human ALK5 (Abcam, Cambridge, UK) was assessed by WB with total proteins from grass carp PBL and HKL. Further confirmation by ICC analysis using grass carp PBL and HKL was also performed. In this experiment, normal rabbit serum (Boster, Wuhan, CH) instead of ALK5 antibody was used as the isotype controls.

### Real-time quantitative PCR (qPCR)

Total RNA from the selected tissues (gill, heart, head kidney, kidney, spleen, liver, intestine, gonad, colla piscis, muscle, TL, HKL, TKL and PBL) was isolated and reverse-transcribed to cDNA using superscript III reverse transcriptase (Invitrogen). The obtained RT samples were used as the template for qPCR. All gene specific primers were listed in Table S1. qPCR amplification was performed on the Bio-Rad CFX96™ Real-time detection system (Bio-Rad, Hercules, USA) in a final volume of 20 µl with 10 µl of IQ™ STBR® Green Supermix (Bio-Rad), 5 µl of diluted (1∶10) RT samples, and 0.2 µM of primers. In these experiments, *β-actin* was also measured as the internal control. qPCR was run for 35 cycles with 20 s at 95°C for denaturation, 20 s at 58°C (*Alk5*, *Igμ* and *Cd8β*) or 60°C (*Ifnγ*, *Tnfα*, *Cd4l*, *Cd8α* and *β-actin*) for annealing and 20 s at 68°C for extension. In order to confirm the specificity of PCR amplification, a melting curve program (60°C–90°C with a heating rate of 0.5°C per second and a continuous fluorescence measurement) was carried out after qPCR. To estimate amplification efficiency, a standard curve was generated for each target gene from 10-fold serial dilution (10^−1^ to 10^−6^ fmole/µl) of the plasmid containing the individual target gene sequence. Each sample was amplified in duplicate.

### Cell viability assay

Leukocyte viability was measured by using Cell Counting Kit-8 (CCK-8, Dojindo, Kumamoto, JP). Briefly, 100 µl of CCK-8 solution was added to the culture medium as the ratio of 1∶10 in each well, and incubated at 27°C for additional 2.5 h. The absorbance was determined by iMark microplate reader (Bio-Rad) at the wavelength of 450 nm with a reference wavelength of 630 nm.

### Western blotting (WB)

Leukocytes (6×10^5^ cells) were collected and lyzed by 0.5% Triton X-100 RIPA lysis buffer (50 mM Tris-HCl, pH 7.5, 1% Triton X-100, 150 mM NaCl, 0.1% SDS, 1 mM DTT and 1 mM EDTA) supplemented with complete EDTA-free Protease Inhibitor Cocktail Tablets (Roche). Protein concentration was determined by the Bradford protein assay (Bio-Rad). The protein samples were separated on 12% (w/v) SDS-PAGE and transferred to the PVDF membrane (Millipore, Bedford, USA) by semidry electro-blotting (Bio-Rad). After blocking for 1 h in TBS/T buffer (10 mM Tris, pH 7.4, 150 mM NaCl,0.05% Tween 20) with 5% (w/v) milk, the membrane strips were incubated with the appropriate primary antibodies [ALK5 pAb (Abcam), 1∶200; TGF-β1 mAb (Abmart), 1∶400; mouse mAb to β-actin, β-actin mAb (Boster), 1∶500] overnight at 4°C, and exposed to horseradish peroxidase (HRP)-conjugated goat anti-rabbit/mouse secondary antibody (Boster) for 1 h at room temperature. Similarly, the specificity of ALK5 pAb was also validated as described in Supplementary Methods. Finally, signals were detected using an ECL kit (Roche) according to the manufacture's instruction.

### Immunocytochemistry (ICC) analysis of grass carp ALK5^+^ leukocytes

Expression of ALK5 was detected by ICC using Histostain™-Plus Kits (ZSGB-BIO, Beijing, CH). Grass carp PBL, HKL or TKL were mounted on poly-L-lysine precoated coverslips and fixed in 4% paraformaldehyde (Sigma-Aldrich). The slips were treated with 3% H_2_O_2_ for 5 min, blocked with goat serum, and then incubated with the 1∶50 diluted ALK5 pAb at 4°C for 16 h. Biotin-labeled goat-anti-rabbit immunoglobulin was added subsequently. The cover slips were washed by 10 mM PBS (pH 7.4) and incubated in streptavidin-HRP solution at 37°C for 30 min. The signals for ALK5 were developed by using a DAB Kit and the cells were afterstained with Harry's hematoxylin (Sigma-Aldrich). The normal rabbit serum (Boster) was used as the isotype control. ALK5^+^ and ALK5^−^ cells were examined with Olympus BX51 microscope (Olympus Optical, Tokyo, JP).

### Data transformation and statistics

Data from CCK-8 assay were expressed as cell viability relative to the respective heat-treated control (*N* = 4) and each experiment was repeated at least three times. The absorbance from control groups were arbitrarily set to 1. In qPCR assay, the value of each sample was the mean of the duplicates in qPCR and the relative mRNA levels of each sample were expressed as mean ± SEM of four individual samples (*N* = 4). The densitometric data of ALK5 protein expression was acquired by Gel Doc EZ system (Bio-Rad) and analyzed with ImageLab software (Bio-Rad). In ICC assay, the number of ALK5^+^ cells was analyzed by AnalySiS Extended Pro 3.1 (Olympus Optical, Tokyo, JP). All data (mean ± SEM) were analyzed with ANOVA followed by Student's *t* test or Fisher's least significance difference (LSD) test. Differences were considered as significant at P<0.05.

## Supporting Information

Figure S1 **Dose-dependent effects of rgcTGF-β1 and ALK5 inhibitor on the viability of PBL, HKL and TKL.** A–B, Leukocytes were incubated with increasing doses (25–400 ng/ml) of rgcTGF-β1, 400 ng/ml of heat treated rgcTGF-β1 or 100 ng/ml of rgcSQT for 72 h. The cell viability of PBL (A, left panel), HKL (A, right panel) and TKL (B) was detected by CCK-8 assay. C, Effects of ALK5 inhibitor on the cell viability of PBL and HKL in the presence of gcTGF-β1. Grass carp PBL or HKL were incubated with 100 ng/ml of native or heat treated rgcTGF-β1 for 72 h in the presence or absence of increasing doses (0.5–8 µM) of ALK5 inhibitor. Relative cell viability was expressed as the fold changes of control group. Results from PBL are presented in the left panels and the right panels were results from HKL. Data presented (mean±SEM, *N* = 4) are representative results of three individual experiments. The alphabet denotes a significant difference at P<0.05.
(TIF)

Figure S2 **Cloning of grass carp** ***Alk5*** **cDNA. A. Full-length cDNA and deduced amino acid sequence of grass carp ALK5.** The coding region is predicted by using Translate tool in ExPASy server. The asterisk (*) indicates the stop codon. The putative polyadenylation signals were marked with black frame. B. Multiple amino acid sequences alignment of grass carp ALK5 with the ALK5s in other species. The activin type I and II receptor domain, GS-motif and serine/threonine protein kinase were noted above the sequences. GenBank accession numbers are as follows: human (NP 004603.1), cattle (NP 777046.1), rat (NP 036907.2), mouse (NP 033396.1), chicken (NP 989577.1), xenopus (NP 001015961.1), zebrafish (NP001032772.2) and grass carp (ADK26459.1). C. Phylogenetic analysis of *Alk5* in vertebrates. The Neighbor-Joining tree was constructed by MEGA3.1 based on the coding sequences of *Alk5* in various vertebrates. The accession numbers are as follows: human (NM_004612.2), cattle (NM_174621.2), rat (NM_012775.2), mouse (NM_009370.2), chicken (D14460.2), xenopus (NM_001015961.2), zebrafish (BC109402.1), and grass carp (HM356028.1). The number at each node indicates the percentage of bootstrapping after 1000 replication. D. Multiple alignment of grass carp ALK5 amino acid sequence with those in other species. The ectodomain, transmembrane region and catalytic domain of kinase were indicated on the sequence. GenBank accession numbers are as follows: human (NP 003233.4), cattle (NP 001153083.1), rat (AAA4237.1), mouse (NP 083851.3), chicken (NP 990759.1), zebrafish (NP 878275.2), salmon (NP 001133728.1) and grass carp (AEK81575.1).
(TIF)

Figure S3 **Validation of the specificity of gcTGF-β1 mAb.** Total protein extracts from grass carp PBL and HKL were used to test the gcTGF-β1 mAb specificity by WB analysis (left panel, lane a, b). Meanwhile, gcTGF-β1 mAb was neutralized by an excess of rgcTGF-β1 (100 µg) to further verify its specificity (right panel, lane a, b).
(TIF)

Figure S4 **Validation of the specificity of ALK5 pAb.** A. Total protein extracts from grass carp PBL and HKL were used to identify the specificity of ALK5 pAb by using WB analysis. B. Confirmation of the ALK5 pAb specificity by ICC assay. The normal rabbit serum was used as the isotype control.
(TIF)

Figure S5 **Effects of TGF-β1 on ALK5 expression and ICC staining of ALK5^+^ cells in grass carp TKL.** After treatment with native or heat treated rgcTGF-β1 (100 ng/ml) for 72 h, ALK5 mRNA (A) and protein (B) levels in TKL were analyzed by qPCR and WB, respectively. Relative mRNA expression levels of *Alk5* were analyzed using *β-actin* as an internal reference and expressed as the fold changes of the heat treated group. Data presented (mean±SEM, *N* = 4) are representative results from three individual experiments. In WB, the representative results were showed and β-actin levels were used as an internal control. Meanwhile, the densitometric analysis of ALK5 protein levels was performed (mean±SEM, *N* = 4) and the relative protein levels were expressed as the fold changes of the heat treated group. C, TKL were treated with 100 ng/ml of native or heat treated rgcTGF-β1 for 72 h, and subsequently fixed on coverslips. After that, ALK5^+^ cells were detected by ICC staining with the ALK5 pAb, and positive cells were observed by using phase contrast microscopy (×400). Representative ICC staining of TKL is presented in left panels and the statistical analysis of the number of ALK5^+^ cells is shown in right panels. The number of ALK5^+^ cells was shown as the percentage of the amount of cells and presented as mean±SEM (*N* = 4).
(TIF)

Table S1 **The primers used in the present study.**
(TIF)
