# Supplementary figures and images for: TGF-β1 Exerts Opposing Effects on Grass Carp Leukocytes: Implication in Teleost Immunity, Receptor Signaling and Potential Self-Regulatory Mechanisms
Source: PLoS One. 2012 Apr 17;7(4):e35011. doi: 10.1371/journal.pone.0035011 (PMC3328490; doi:10.1371/journal.pone.0035011)

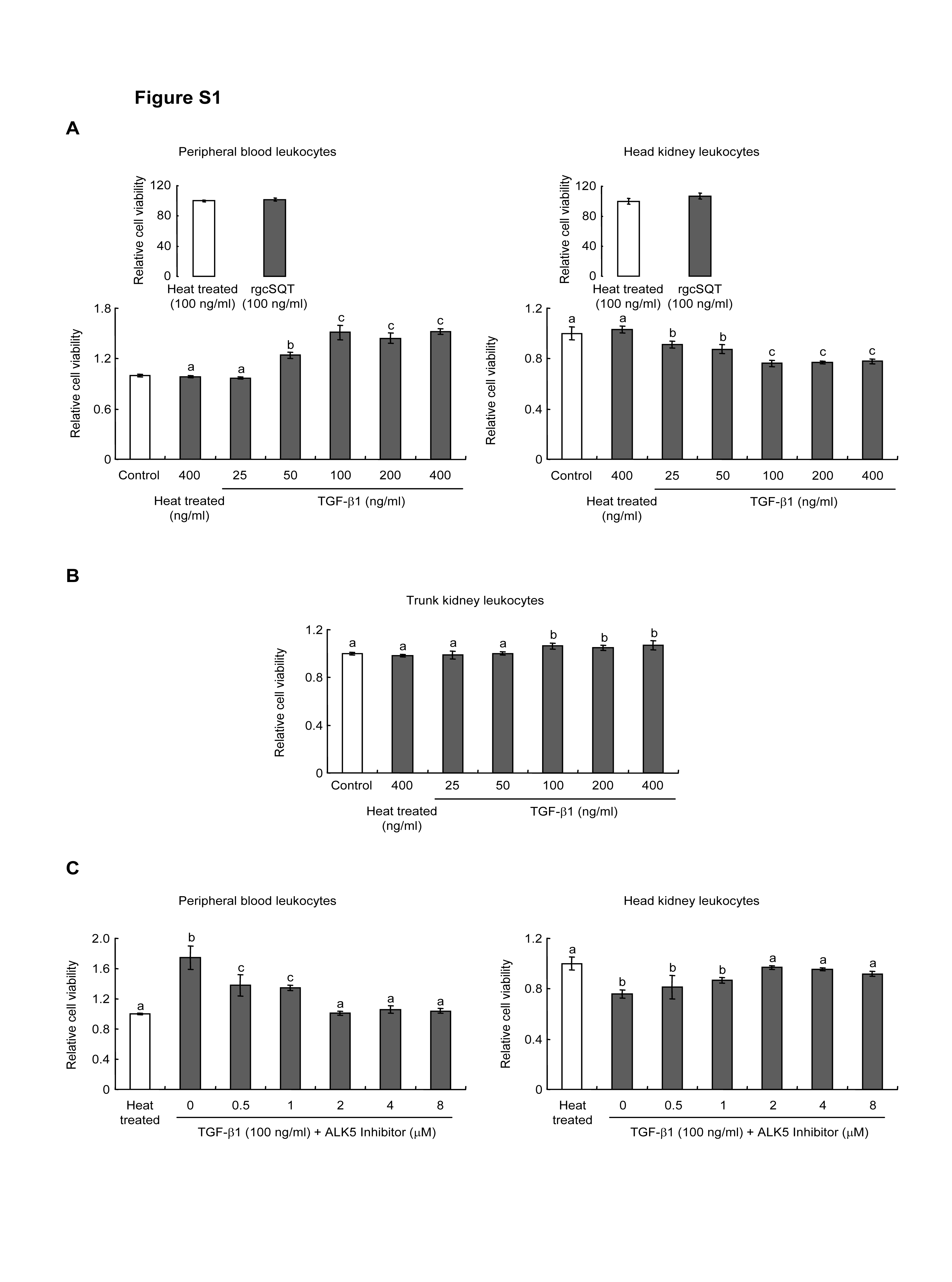

Supplement: Figure S1 — Dose-dependent effects of rgcTGF-β1 and ALK5 inhibitor on the viability of PBL, HKL and TKL. A–B, Leukocytes were incubated with increasing doses (25–400 ng/ml) of rgcTGF-β1, 400 ng/ml of heat treated rgcTGF-β1 or 100 ng/ml of rgcSQT for 72 h. The cell viability of PBL (A, left panel), HKL (A, right panel) and TKL (B) was detected by CCK-8 assay. C, Effects of ALK5 inhibitor on the cell viability of PBL and HKL in the presence of gcTGF-β1. Grass carp PBL or HKL were incubated with 100 ng/ml of native or heat treated rgcTGF-β1 for 72 h in the presence or absence of increasing doses (0.5–8 µM) of ALK5 inhibitor. Relative cell viability was expressed as the fold changes of control group. Results from PBL are presented in the left panels and the right panels were results from HKL. Data presented (mean±SEM, N = 4) are representative results of three individual experiments. The alphabet denotes a significant difference at P<0.05. (TIF) [file pone.0035011.s001.tif]

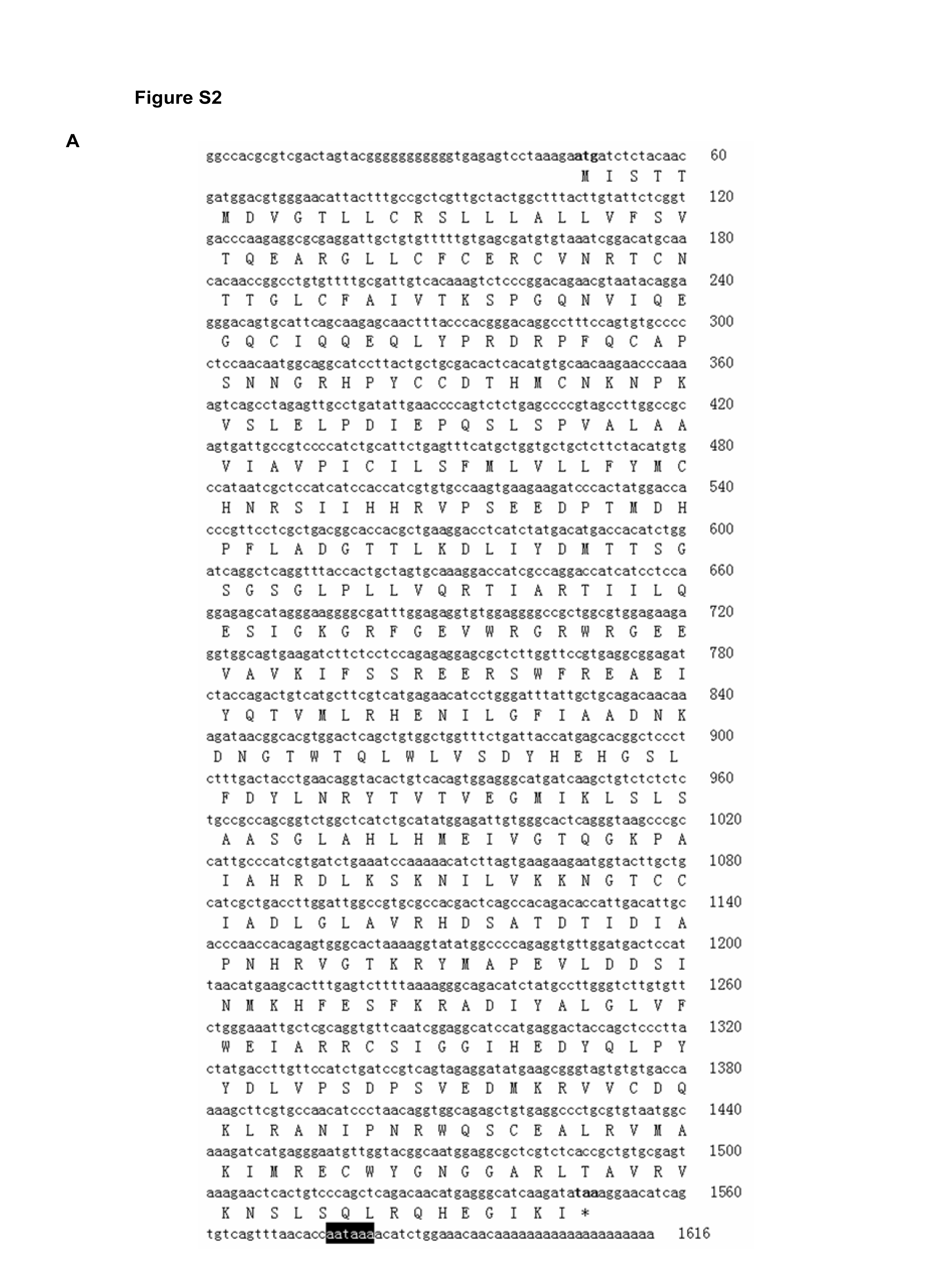

Supplement: Figure S2 — Cloning of grass carp Alk5 cDNA. A. Full-length cDNA and deduced amino acid sequence of grass carp ALK5. The coding region is predicted by using Translate tool in ExPASy server. The asterisk (*) indicates the stop codon. The putative polyadenylation signals were marked with black frame. B. Multiple amino acid sequences alignment of grass carp ALK5 with the ALK5s in other species. The activin type I and II receptor domain, GS-motif and serine/threonine protein kinase were noted above the sequences. GenBank accession numbers are as follows: human (NP 004603.1), cattle (NP 777046.1), rat (NP 036907.2), mouse (NP 033396.1), chicken (NP 989577.1), xenopus (NP 001015961.1), zebrafish (NP001032772.2) and grass carp (ADK26459.1). C. Phylogenetic analysis of Alk5 in vertebrates. The Neighbor-Joining tree was constructed by MEGA3.1 based on the coding sequences of Alk5 in various vertebrates. The accession numbers are as follows: human (NM_004612.2), cattle (NM_174621.2), rat (NM_012775.2), mouse (NM_009370.2), chicken (D14460.2), xenopus (NM_001015961.2), zebrafish (BC109402.1), and grass carp (HM356028.1). The number at each node indicates the percentage of bootstrapping after 1000 replication. D. Multiple alignment of grass carp ALK5 amino acid sequence with those in other species. The ectodomain, transmembrane region and catalytic domain of kinase were indicated on the sequence. GenBank accession numbers are as follows: human (NP 003233.4), cattle (NP 001153083.1), rat (AAA4237.1), mouse (NP 083851.3), chicken (NP 990759.1), zebrafish (NP 878275.2), salmon (NP 001133728.1) and grass carp (AEK81575.1). (TIF) [file pone.0035011.s002.tif]

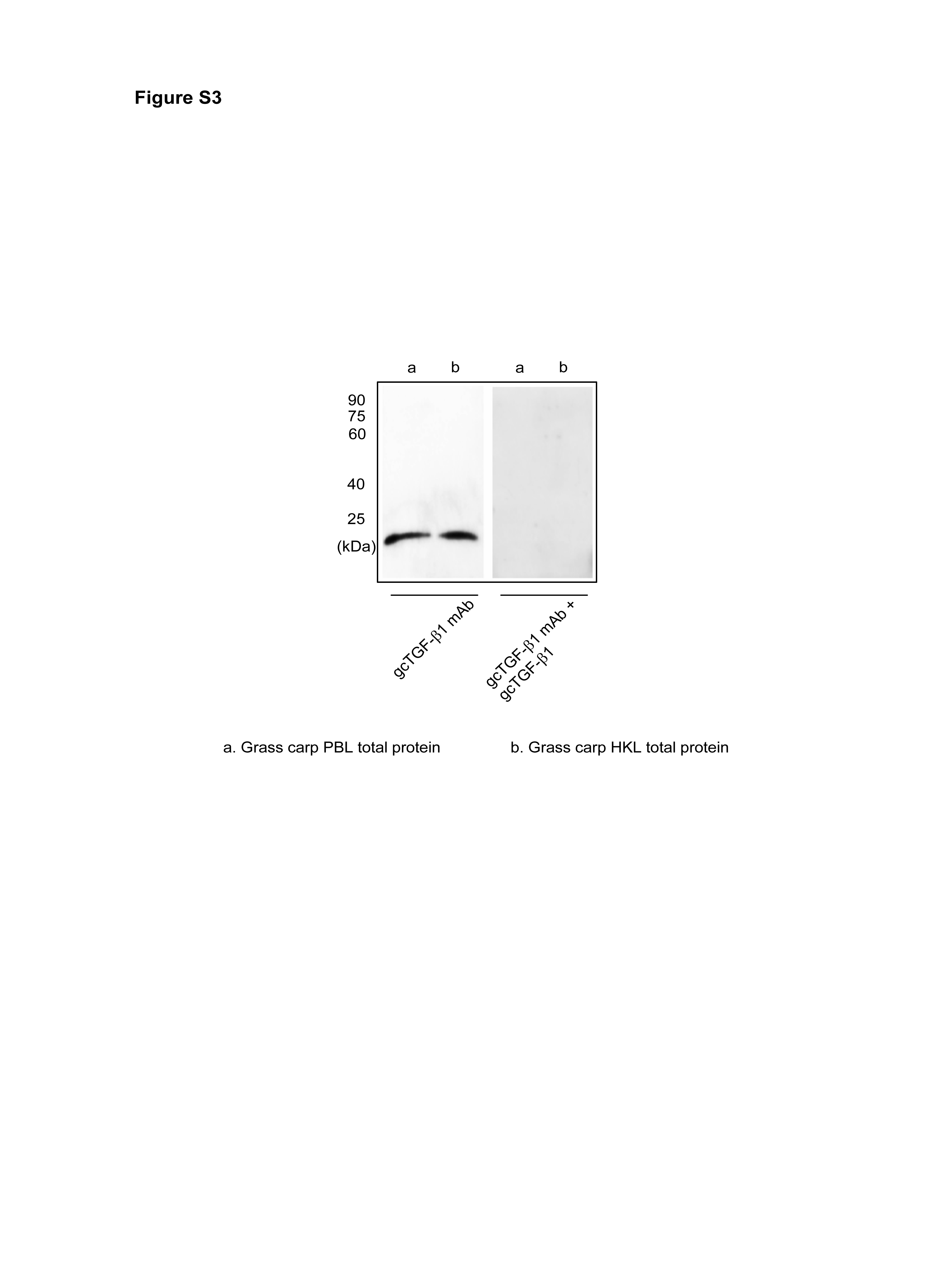

Supplement: Figure S3 — Validation of the specificity of gcTGF-β1 mAb. Total protein extracts from grass carp PBL and HKL were used to test the gcTGF-β1 mAb specificity by WB analysis (left panel, lane a, b). Meanwhile, gcTGF-β1 mAb was neutralized by an excess of rgcTGF-β1 (100 µg) to further verify its specificity (right panel, lane a, b). (TIF) [file pone.0035011.s003.tif]

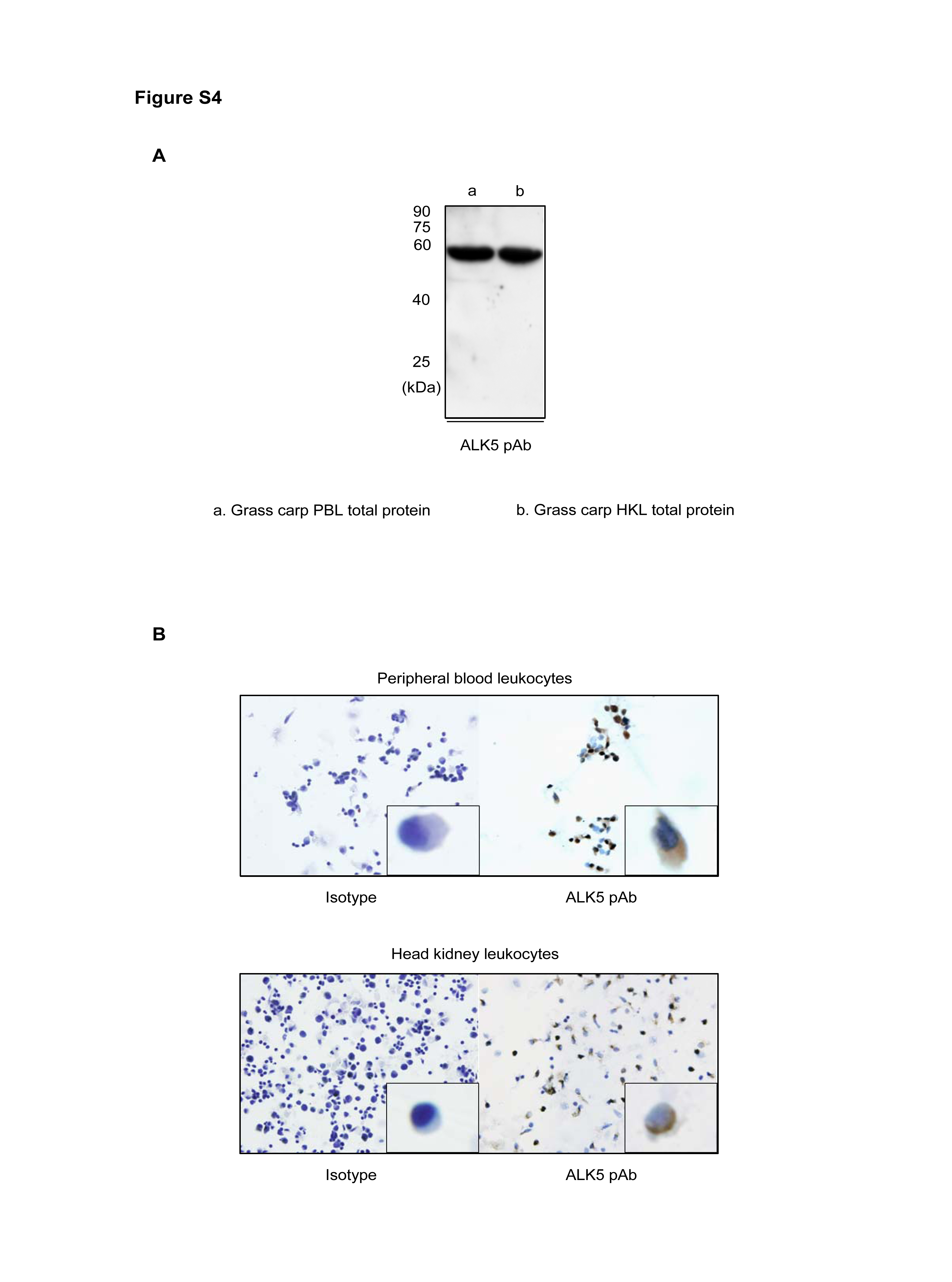

Supplement: Figure S4 — Validation of the specificity of ALK5 pAb. A. Total protein extracts from grass carp PBL and HKL were used to identify the specificity of ALK5 pAb by using WB analysis. B. Confirmation of the ALK5 pAb specificity by ICC assay. The normal rabbit serum was used as the isotype control. (TIF) [file pone.0035011.s004.tif]

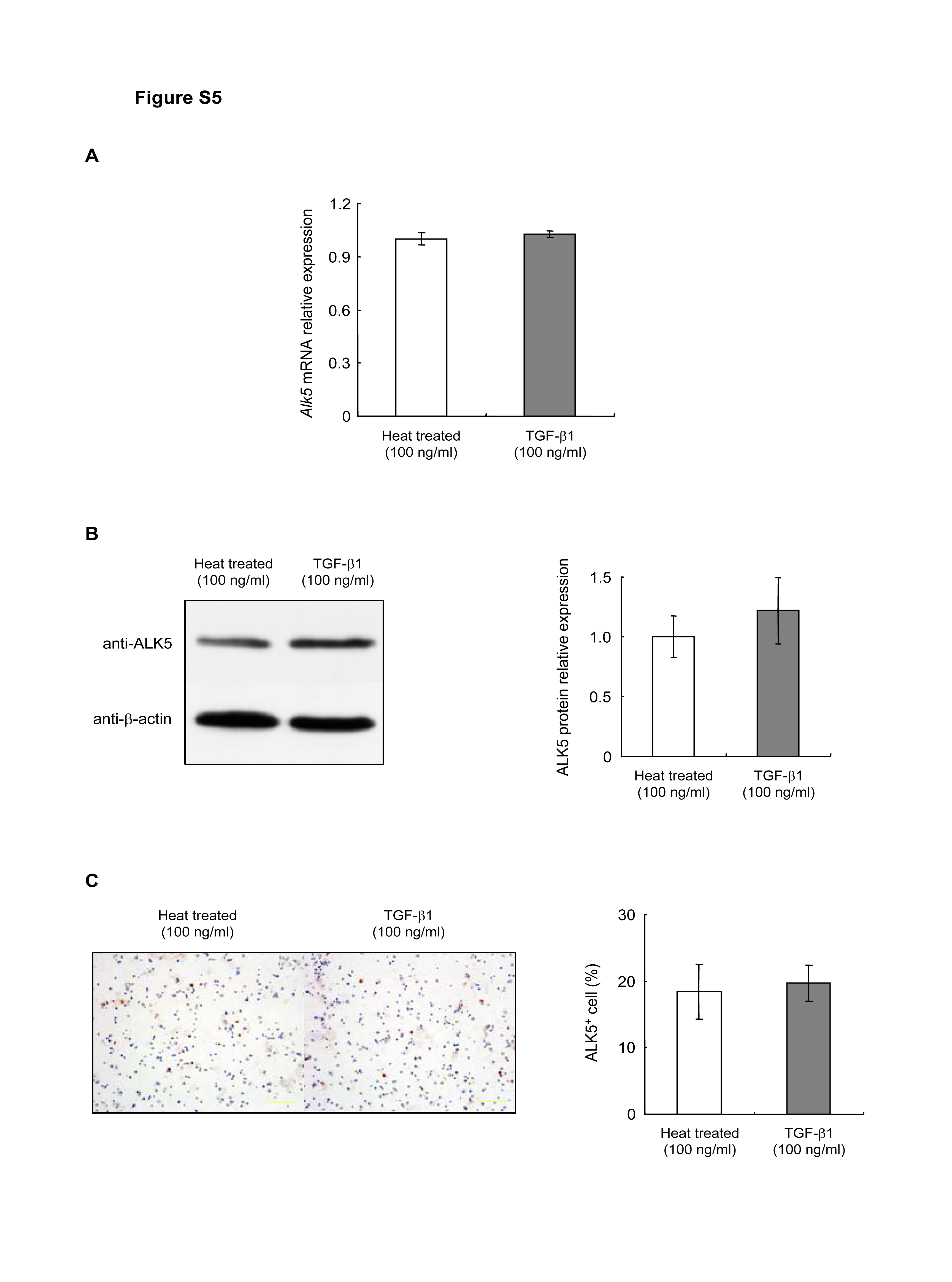

Supplement: Figure S5 — Effects of TGF-β1 on ALK5 expression and ICC staining of ALK5+ cells in grass carp TKL. After treatment with native or heat treated rgcTGF-β1 (100 ng/ml) for 72 h, ALK5 mRNA (A) and protein (B) levels in TKL were analyzed by qPCR and WB, respectively. Relative mRNA expression levels of Alk5 were analyzed using β-actin as an internal reference and expressed as the fold changes of the heat treated group. Data presented (mean±SEM, N = 4) are representative results from three individual experiments. In WB, the representative results were showed and β-actin levels were used as an internal control. Meanwhile, the densitometric analysis of ALK5 protein levels was performed (mean±SEM, N = 4) and the relative protein levels were expressed as the fold changes of the heat treated group. C, TKL were treated with 100 ng/ml of native or heat treated rgcTGF-β1 for 72 h, and subsequently fixed on coverslips. After that, ALK5+ cells were detected by ICC staining with the ALK5 pAb, and positive cells were observed by using phase contrast microscopy (×400). Representative ICC staining of TKL is presented in left panels and the statistical analysis of the number of ALK5+ cells is shown in right panels. The number of ALK5+ cells was shown as the percentage of the amount of cells and presented as mean±SEM (N = 4). (TIF) [file pone.0035011.s005.tif]

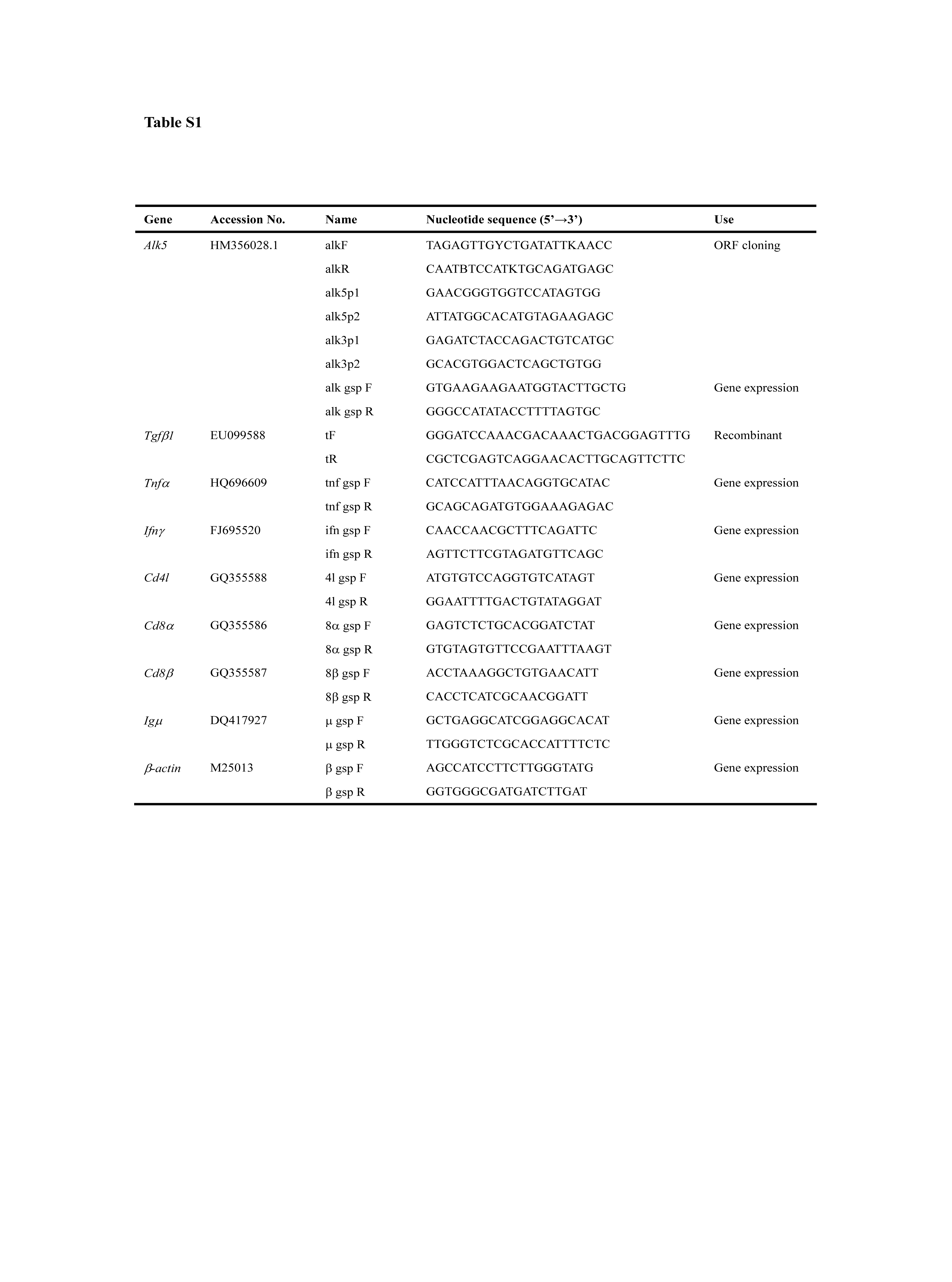

Supplement: Table S1 — The primers used in the present study. (TIF) [file pone.0035011.s006.tif]
